# An open source chemical structure curation pipeline using RDKit

**DOI:** 10.1186/s13321-020-00456-1

**Published:** 2020-09-01

**Authors:** A. Patrícia Bento, Anne Hersey, Eloy Félix, Greg Landrum, Anna Gaulton, Francis Atkinson, Louisa J. Bellis, Marleen De Veij, Andrew R. Leach

**Affiliations:** 1 European Molecular Biology Laboratory, European Bioinformatics Institute, Wellcome Genome Campus, Hinxton, CB10 1SD Cambridgeshire UK; 2 T5 Informatics GmbH, Basel, 4055 Switzerland; 3 Present Address: The Cambridge Crystallographic Data Centre, 12 Union Road, Cambridge, CB2 1EZ UK; 4 Present Address: Department of Oncology, University of Cambridge, Cambridge, UK

**Keywords:** Chemistry, Curation, ChEMBL, RDKit, Open source, Standardisation

## Abstract

**Background:**

The ChEMBL database is one of a number of public databases that contain bioactivity data on small molecule compounds curated from diverse sources. Incoming compounds are typically not standardised according to consistent rules. In order to maintain the quality of the final database and to easily compare and integrate data on the same compound from different sources it is necessary for the chemical structures in the database to be appropriately standardised.

**Results:**

A chemical curation pipeline has been developed using the open source toolkit RDKit. It comprises three components: a *Checker* to test the validity of chemical structures and flag any serious errors; a *Standardizer* which formats compounds according to defined rules and conventions and a *GetParent* component that removes any salts and solvents from the compound to create its parent. This pipeline has been applied to the latest version of the ChEMBL database as well as uncurated datasets from other sources to test the robustness of the process and to identify common issues in database molecular structures.

**Conclusion:**

All the components of the structure pipeline have been made freely available for other researchers to use and adapt for their own use. The code is available in a GitHub repository and it can also be accessed via the ChEMBL Beaker webservices. It has been used successfully to standardise the nearly 2 million compounds in the ChEMBL database and the compound validity checker has been used to identify compounds with the most serious issues so that they can be prioritised for manual curation.
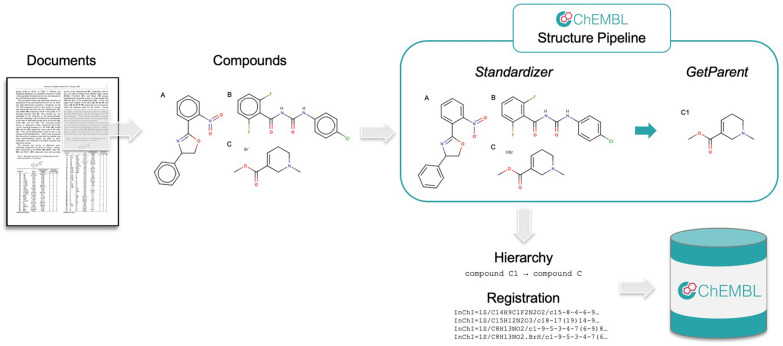

## Introduction

The ChEMBL database is a freely available bioactivity database containing close to 2.5 million compound records on nearly 2 million unique chemical structures [1]. The compound structures and associated bioactivity data are extracted on a regular basis primarily from the medicinal chemistry literature. A growing number of researchers are also depositing experimental data directly in order to make these available in the public domain. Furthermore, ChEMBL contains a set of manually curated marketed drugs and clinical candidates as well as selected bioactivity data from other public databases such as BindingDB [2] and PubChem [3]. Bioactivity data on the same compound from all ChEMBL sources (scientific articles, deposited datasets and curated drug sources) are aggregated according to chemical structure. Compounds may be physically tested in bioassays as the so-called parent molecule or as one of a number of different salt forms. Scientists commonly wish to aggregate the data on these different forms on the basis of the common underlying parent structure, and so it is necessary to link these various forms of the “same” underlying parent molecule.

In order to facilitate the use of the database, a key objective of the ChEMBL compound curation process is to standardise the chemical structures stored in the database and to assign a unique identifier to each distinct chemical structure regardless of the source. It is worth noting that there are over 5000 unique compounds in the ChEMBL database with data from ten or more different sources, and four compounds (doxorubicin, ciprofloxacin, chloroquine and paclitaxel) each with data from over 1000 sources. For each ChEMBL release, more than 50,000 new structures are added to the database, which makes manual curation and standardisation of the chemical structures impracticable. Hence an automated procedure is required.

The chemical structures submitted to the ChEMBL database are generally received as molfiles [4] but can also be in SMILES format [5]. There is no universal standard for these formats and the challenges of converting between chemical structure formats is well documented [6]. Even the simple process of loading molecules into and out of different cheminformatics packages can subtly change the structure, particularly if it was not well drawn in the first place. Chemical structures from the primary scientific literature are mostly manually drawn from the structural information in the papers prior to loading into ChEMBL. These structures are often represented in the publication as Markush structures with different R-groups shown in SAR (structure–activity relationship) tables. Compounds may also be shown with charges on acidic or basic groups, to indicate the form in which they are likely to interact with amino acid residues in a binding pocket. In some articles the compounds synthesised and reported are isotopically labelled. The ChEMBL compound curation procedure therefore needs to process molecules represented in all these ways (and more) to determine which compounds are the same. Examples of these situations can be found in recent articles where data was extracted for the ChEMBL database [7–9].

A standardised V2000 molfile was chosen as the primary chemical structure representation in the database. The Standard InChI and the corresponding hashed InChIKey [10, 11] are used in ChEMBL as the measure of uniqueness for a chemical structure and are calculated from the molfile using software provided by the InChI Trust, currently version 1.05 [12]. Thus, when compounds from different scientific articles have the same Standard InChI and InChIKey they are considered to be the same compound and are assigned the same ChEMBL identifier (CHEMBL_ID). The use of the Standard InChI has a number of advantages in the context of the ChEMBL database. It was developed as an IUPAC open standard to enable information exchange and interoperability between large databases. The simple format of Standard InChI is also used by many other database providers and hence it is an ideal choice for an open database such as ChEMBL. However, unlike molfiles and SMILES, it was designed as an identifier and not as a structure format suitable for cheminformatics applications. For example, the Standard InChI is independent of the tautomeric form of a compound and hence different tautomers of a compound will have the same standard InChI. As a consequence, they are considered in ChEMBL as being the same compound and hence have the same ChEMBL_ID. It should however be remembered that there are a few disadvantages to using Standard InChI as an identifier, including: its inability to recognise some 1,5 keto-enol tautomers as being the same compound; its inability to recognise cis/trans isomerism in organometallic compounds (e.g. cisplatin and transplatin) and it does not support the use of relative stereochemistry, only absolute or no stereochemistry. Despite these limitations, it is a good compromise for a structural identifier for a public database such as ChEMBL. To aid interoperability, a canonical SMILES is also generated from each primary molfile, but this is considered to be a secondary structure.

The challenges of registering compounds in a database and determining chemical uniqueness are not specific to ChEMBL. PubChem, the open chemistry database of over 100 million compounds maintained by the National Institutes of Health (NIH) has developed an automated and publicly available standardisation process based on OpenEye toolkits [13]. They utilise an isomeric canonical SMILES to identify unique structures and show that their method generates more unique structures than when Standard InChI is used to determine uniqueness. They also generate a canonical tautomer for their compounds. The ChEMBL group decided against the use of a canonical tautomer, for a number of reasons. Firstly, it is anticipated that the authors of an article in the medicinal chemistry literature will have worked on their compound set for some considerable time, performed docking and molecular modelling studies and will be well placed to assign the most appropriate tautomeric form, for example based on specific interactions with the target protein. Secondly, it is possible for tautomers to interconvert under experimental conditions. Thirdly, changing the tautomeric form of a compound can alter its stereochemistry by removing, or introducing, a chiral centre from a molecule, requiring often difficult decisions to be made about how to “merge” compounds/data with different chirality or which stereo form to create. Thalidomide is a well-known example of the second phenomenon which interconverts between the therapeutic R-enantiomer and the teratogenic S-enantiomer via the enol tautomer [14].

The Royal Society of Chemistry (RSC) developed the Chemical Validation and Standardization Platform (CVSP) [15] to support compound deposition into their ChemSpider chemical database [16] and as a contribution to the Innovative Medicines Initiative (IMI) funded OpenPHACTS project [17] which aimed to standardise chemical structures from multiple databases. The CVSP methodology uses sets of SMARTS-based rules that can be tailored by the user. The code is available on GitHub [18] but currently has no obvious documentation and the interface is no longer available [19] although it is still possible for depositors to ChemSpider to use the rules to validate their structures.

The United States Environmental Protection Agency (EPA) also strive to have well curated structures in their DSSTox database [20]. They have described, in detail, the complexity of the task and have undertaken extensive curation of their chemical structures using a combination of manual and automated methods. Much of their focus has been on resolving the mismatches between names, identifiers and structures between their compound set and those in the US National Library of Medicine databases ChemID [21] and PubChem. Due to the inability of the V2000 molfiles to distinguish relative and absolute stereochemistry they have chosen to use V3000 molfiles as their preferred structure format. They also use an InChIKey calculated using the ChemAxon JChem toolkit to determine uniqueness, but this differs from the Standard InChIKey discussed above. SMILES are provided for users not wanting to use V3000 molfiles, but they make the point that this results in a less rich representation of the structures. Their methods are based on a combination of commercial toolkits and their own manual curation tool [22].

The Global Substance Registration System (GSRS), developed by the regulatory authorities in the USA and Europe, creates a Unique Ingredient Identifier (UNII) for components of medicinal products which includes small molecules but also more complex molecules such as proteins and polymers [23]. This is a particularly useful resource for identifying the structures of specific pharmaceutically relevant compounds, but it is less useful for the bulk curation of the larger set of bioactive molecules in the ChEMBL database.

Many commercial software providers also provide toolkits for standardising compound structures. These are widely used by large pharmaceutical and agrochemical companies where the precise annotation of a chemical structure is crucial for intellectual property protection. Those produced by ChemAxon and 3DS are examples of these toolkits [24, 25].

The legacy pipeline for processing compound structures prior to deposition into ChEMBL was based on commercial software and had evolved over more than 10 years. During this time, more and more incorrect, unusual, and exceptional situations were identified and integrated into the pipeline. Unfortunately, these refinements were increasingly making the code difficult to maintain and modify. In reviewing options for a sustainable future solution that also removed the dependence on commercial software it became apparent that none of the existing toolkits fitted the ChEMBL group’s requirements. Therefore, the decision was made to build a curation pipeline around the widely used open-source RDKit toolkit and its implementation of the MolVS molecule validation and standardisation tool [26, 27]. The resulting pipeline is now available as an open-source solution, freely available for the wider scientific community [28]. As part of this project the current rules used for curating ChEMBL structures have also been revised and rewritten to make them more sustainable. The resulting processes are designed and developed to suit the specific situation of ChEMBL, but the code can also be used by others “as is” or can be modified to suit other requirements.

It is important to note that this curation pipeline is not intended as a replacement for a similarly named standardiser tool [29] which was previously developed by the ChEMBL group as a contribution to the IMI eTOX project [30] and was specifically designed to standardise molecules in preparation for molecular modelling applications.

The newly developed curation pipeline will now be described in more detail.

## Methods

Three new components have been developed using the RDKit toolkit. Two of these components (*Standardizer* and *GetParent*) have been rewritten and adapted from rules originally implemented using a commercial software toolkit. In contrast, the *Checker* component was developed more recently in an attempt to identify problem structures before they were added to the ChEMBL database.

Hence the new ChEMBL curation pipeline comprises three processes:

1. *Checker*: identifies and validates structures and identifies problems before they are added to the database
2. *Standardizer*: processes (standardises) chemical structures according to a set of predefined rules
3. *GetParent*: generates parent structures based on a set of rules and defined lists of salts and solvents

### *Checker* component

The *Checker* component validates structures prior to the compounds being loaded into ChEMBL. If an error or problem is detected in the structure a score is reported for the molecule; the score increases with the severity of the perceived problem. In the majority of cases compounds are loaded into the database even if a warning flag is identified. The scores are recorded but at this point errors are not corrected. Instead, they are prioritised and subjected to subsequent manual curation, as time and degree of seriousness permits. A summary of the structure checks performed, and the resultant penalty scores assigned are shown in Table 1.

**Table 1:** Penalty scores and annotation that are output from the *Checker* module

Penalty score	Penalty explanation
7	Error-9986 (Cannot process aromatic bonds) Illegal input InChI: Unknown element(s)
6	All atoms have zero coordinates InChI: Accepted unusual valence(s) InChI: Empty structure Molecule has 3D coordinates Molecule has a radical that is not found in the known list Molecule has six (or more) atoms with exactly the same coordinates Number of atoms less than 1 Polymer information in mol file V3000 mol file
5	InChI_RDKit/Mol stereo mismatch Mol/Inchi/RDKit stereo mismatch RDKit_Mol/InChI stereo mismatch Molecule has a bond with an illegal stereo flag Molecule has a bond with an illegal type Molecule has a crossed bond in a ring Molecule has two (or more) atoms with exactly the same coordinates
2	InChI_Mol/RDKit stereo mismatch Molecule has a stereo bond in a ring Molecule has an atom with multiple stereo bonds Molecule has a stereo bond to a stereocenter Molecule has the 3D flag set for a 2D conformer Other InChI Warnings

7 is the most serious penalty score and 2 the least important

It is an individual user’s choice what they decide to do with molecules that return specific penalty scores. For ChEMBL, a penalty score of 7 is considered to be a fatal error and the molfile is not loaded into the database. Examples of illegal input are, for example, unknown elements in the molfile, or molfiles that cannot be read in RDKit due to the inability to process their aromatic bonds. Molecules with a penalty score of 6 are loaded into the ChEMBL database but without a molfile, as it is considered that these have a significant issue with the structure, and it is preferable to fix the problem than have a badly formed molecule in the database. Most of the issues that give rise to a penalty score of 6 are self-explanatory and are described in Table 1. If the penalty score is 5 or 2 the molecule is loaded but the compounds are also prioritised for manual curation. Again, many of the 5 and 2 penalty scores are self-explanatory, but the stereo mismatch errors perhaps need further explanation. These are reported when the number of stereocentres perceived by the following calculation methods differ:

- Mol: number of atoms where a wedged bond starts
- InChI: number of tetrahedral stereocentres
- RDKit: number of atomic stereocentres remaining after calling Chem.AssignStereochemistry()

Hence the “InChi_RDKit/Mol stereo mismatch” warning message indicates that the InChI and RDKit algorithms perceive the number of stereocentres to be the same but different from the molfile. “Mol/Inchi/RDKit stereo mismatch” means that all three methods perceive different stereocentre counts. The majority of these issues occur in complex molecules such as bridged bicyclic molecules that are badly drawn. As Standard InChI is derived from the molfile, the errors where the molfile and InChI differ in their stereocentre counts are given a higher penalty score [5] than when they are the same but different from the RDKit stereo count [2].

The InChI software may give a number of warnings. These are also reported by the ChEMBL *Checker* module. Some of these are considered important, but others such as “InChI: Omitted undefined stereo”, “InChI: Charges were rearranged”, “InChI: Ambiguous stereo”, “InChI: Proton(s) added/removed” and “InChI: Not chiral” are generated for large numbers of molecules. These either reflect the reorganisation of atoms in order to generate the InChI or are related to stereochemical ambiguity arising, for example, from the fact that the compound is a racemate; these are not considered issues for a database such as ChEMBL. Therefore, these are given a low penalty score [2]. However, in other contexts they might be more relevant and so are reported in the *Checker* output.

### *Standardizer* component

The standardisation rules implemented in the ChEMBL database are based largely on the FDA/IUPAC guidelines [31, 32]. Whilst the aim is to adhere to these rules as closely as possible, the practical reality is that submitted compounds are sometimes drawn imperfectly or the structures are ambiguously defined in the original publication or by the depositor. An automated standardiser can only safely correct some of the potential issues and the standardisation rules, currently encoded in the *Standardizer* component, are outlined here.

For certain compound types, particularly organometallic and those with a large number of boron atoms, a flag is set (exclude flag) and no attempt is made to standardise them. This is largely due to the V2000 molfile format used by ChEMBL being unable to accurately represent coordination bonds. For this reason, although the bioactivity data on these compounds is available in ChEMBL, the chemical structures are not curated nor provided in the release version of the database.

The first step in the standardisation process is therefore to exclude molecules if they contain more than 7 boron atoms or any of the following atoms: [Sc], [Ti], [V], [Cr], [Mn], [Fe], [Co], [Ni], [Cu], [Ga], [Y], [Zr], [Nb], [Mo], [Tc], [Ru], [Rh], [Pd], [Cd], [In], [Sn], [La], [Hf], [Ta], [W], [Re], [Os], [Ir], [Pt], [Au], [Hg], [Tl], [Pb], [Bi], [Po], [Ac], [Ce], [Pr], [Nd], [Pm], [Sm], [Eu], [Gd], [Tb], [Dy], [Ho], [Er], [Tm], [Yb], [Lu], [Th], [Pa], [U], [Np], [Pu], [Am], [Cm], [Bk], [Cf], [Es], [Fm], [Md], [No], [Lr], [Ge], [Sb].

The following standardisations are then made to the molecule (where they occur):

1. Standardise unknown stereochemistry
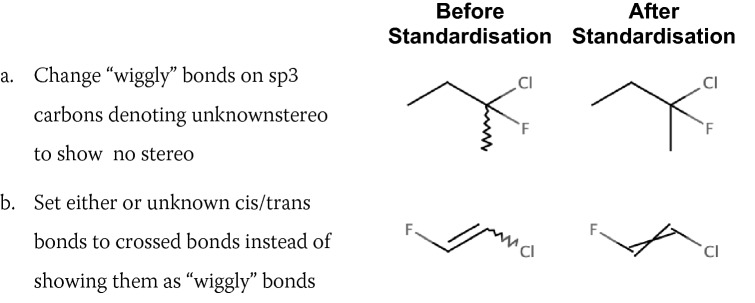
2. Clear S Group data from the molfile
3. Generate a kekulé form of the structure
4. Remove explicit H atoms except:
  - Hs where an isotope of hydrogen has been specifically set
  - Hs that have a wedged or dashed bond to them
  - Hs bonded to atoms with tetrahedral stereochemistry set (“Chiral Hs”). This is an example:
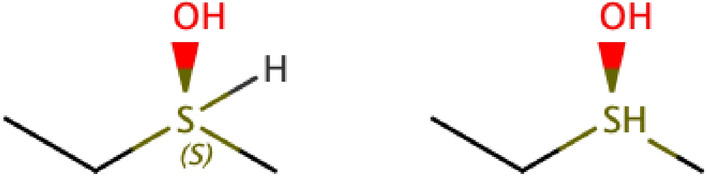
  - Hs bonded to atoms in a non-default valence state that are not simply protonated. An example is phosphinic acid:
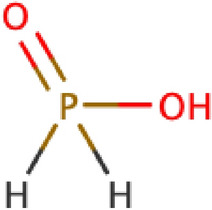
5. Normalise structure:
  - Fix hypervalent nitro groups
  - Convert covalently drawn alkaline metals connected to O or N to ionic forms (e.g. NaO to Na+ O−)
  - Fix incorrect amide tautomers, e.g. N=COH to HNC(=O)
  - Standardise sulphoxides to charge-separated form
  - Standardise diazonium N to N+
  - Ensure quaternary N is charged
  - Ensure trivalent O is charged
  - Ensure trivalent S is charged
  - Ensure halogen not bonded to a neighbouring atom is charged
6. Ensure molecule is neutralised, if possible, by:
  - Adding or removing Hs
  - Moving Hs from one atom to another (including between components)

Note that if the Hs could be added to more than one atom an arbitrary choice is made but this is done canonically so the result will always be the same for a given molecule

7. Normalise (straighten) triple bonds and allenes

In the context of the ChEMBL database, it is the molfiles standardised according to these rules that are stored in the database and which are in turn the structures made available to the database users.

### *GetParent* component

Many compound registration systems, including the ChEMBL database, identify compounds that are related by virtue of being a salt form of a common parent structure. Therefore, as part of the ChEMBL compound curation pipeline, molecules are identified where the molfile contains more than one connected component as well as molecules containing atoms with specified isotopes.

The *GetParent* module is applied to just those compounds that match one or both of these criteria. All information about isotopes is removed, as are solvents and salts present in the molfile which match any of the components in the defined salt and solvent lists. Having removed all salts (e.g. Na+ that might be included to neutralise a carboxyl group), the resulting molecule is neutralised and a new molfile created as the “parent” molecule. Compounds containing more than one component that are genuine mixtures (i.e., all of the components are absent from the salt and solvent lists) has, in the context of the ChEMBL database, its parent registered as the identical mixture. For cases such as sodium chloride and sodium citrate, where both components are in the salt list, the *GetParent* module does not remove any component and the parent remains the same as the salt. Here again, the parent is registered as the multicomponent mixture. Compounds containing any of the excluded atoms described above have their isotopes and solvents removed and then parents created, so that bioactivity data can subsequently be aggregated. For example, the antimony-containing compound sodium stibogluconate has two versions in ChEMBL 26, both with bioactivity data: CHEMBL3754364 is a version with water of crystallisation and CHEMBL3764926 is a version without. These are annotated as related forms so that the bioactivity data can be seen aggregated in the database. Cyanocobalamin is a cobalt-containing compound which is recorded as a parent and three different isotopes in the database (CHEMBL2110563, CHEMBL2104118, CHEMBL2104381 and CHEMBL2096655). Again, the *GetParent* module enables their data aggregation. Organometallic compounds do not however have salts removed due to the complexity of how they are often represented in the deposited molfile. For example, this is often achieved by drawing them as disconnected components as is the case for transplatin (CHEMBL1386) which was deposited into the database as N.N.[Cl−].[Cl−].[Pt+2]. Removal of the chloride and ammonia components would incorrectly result in a platinum ion as the parent.

The list of salts used in ChEMBL is based on the USAN Council’s list of pharmacological salts [33]. Additional entities have been added to this list where a significant number of examples have been present in ChEMBL datasets. The *GetParent* module will remove salts regardless of: (i) the charge status (e.g. acetic acid or acetate, Cl− or HCl); (ii) whether or not stereochemistry is depicted (e.g. tartaric acid); (iii) cis/trans isomers (e.g. maleic and fumaric acid). The salts and solvents files are available in the GitHub repository [28]. Currently, these files contain 162 salts and 9 solvents respectively. This list will be maintained and extended if additional salts and solvents are identified.

**Fig. 1:**
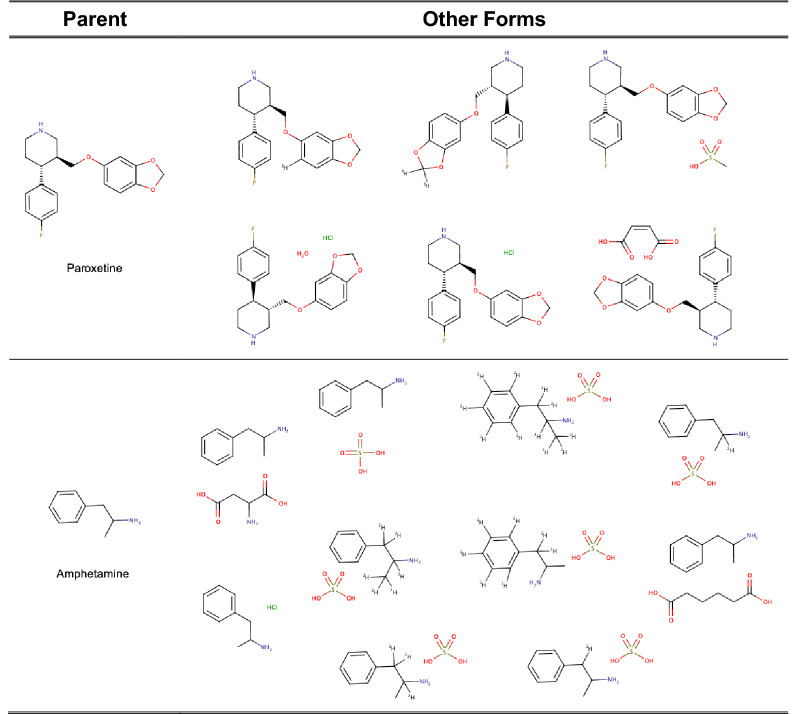
Examples of the multicomponent forms of paroxetine and amphetamine and how they have been aggregated by use of the *GetParent* component

For the avoidance of any doubt, although parents, salts, solvents, isotopes and mixtures are all identified using the process just described, the bioactivity data recorded in ChEMBL is registered against the form it was measured on. The aggregation by parent structure is undertaken to make it easier to identify all the data for salts and isotopes of a common parent. For example, paroxetine (CHEMBL490) has bioactivity data determined for the parent molecule, two salts, one salt/solvent mixture and two different isotopes as well as there being an additional salt registered as an FDA approved drug. Another example is amphetamine (CHEMBL405), which has bioactivity data in ChEMBL for eight different salts in addition to the parent amphetamine and an additional two salts that are recorded in drug sources such as the FDA orange book [34]. The parent aggregation process makes this data easily identified and grouped. This is illustrated in Fig. 1 for these two compounds.

### Availability of structure curation pipeline

The code for the pipeline has all been developed using the RDKit toolkit (version 2019.09.2.0). It is open source and publicly available in GitHub [28], currently as version 1.0.0. A conda package is also available to facilitate installation [35]. The *Standardizer*, *Checker* and *GetParent* functions are also integrated in the ChEMBL Beaker webservices and can be used in this way via the ‘check’, ‘getParent’ and ‘standardize’ endpoints [36]. Any new features developed by the ChEMBL group will be added to the repository and comments and suggestions from others are welcomed.

## Results and discussion

One of the objectives in developing the new curation pipeline was to improve the quality of the chemical structures in the ChEMBL database. As the rules implemented in the new *Standardizer* and *GetParent* modules were largely based on the original ChEMBL rules developed over the last 10 years it is difficult to unambiguously quantify this improvement. To provide a consistent starting point, therefore, the current 1.9 million compounds in the latest release of ChEMBL (ChEMBL 26) have been completely re-standardised and the parents regenerated for the whole database using the new pipeline.

### *Checker* component

To investigate the utility of the *Checker* module three very different compound sets were used. These were: a set of compounds recently extracted (by manual drawing) from the medicinal chemistry literature by the ChEMBL data extractors; a set of compounds from SureChEMBL, the patent database where the chemical structures are obtained by entity recognition followed by name or image to structure conversion [37] and a randomly selected compound set from PubChem (SIDs) as initially submitted by the depositors and prior to any compound standardisation. These three datasets are provided as structure data files (sd files) in the Supplementary Information S1, S2, S3. The results from these three sets are shown in Table 2.

**Table 2:** *Checker* total number of the different penalty scores output from subjecting the ChEMBL Literature set, the SureChEMBL set and the PubChem Set to the *Checker* process

Penalty score	Penalty explanation	SureChEMBL	ChEMBL Literature	PubChem
7	Error-9986 (Cannot process aromatic bonds)	4	0	0
	Illegal input	0	1	0
	InChI: Unknown element(s)	3	0	1355
6	All atoms have zero coordinates	0	0	12
	InChI: Accepted unusual valence(s)	73	1	2155
	InChI: Empty structure	0	1	5824
	Molecule has 3D coordinates	0	1	1024
	Molecule has a radical that is not found in the known list	187	1	252
	Molecule has six (or more) atoms with exactly the same coordinates	3	0	206
	Number of atoms less than 1	0	1	5824
	Polymer information in mol file	2	0	0
	V3000 mol file	594	0	0
5	InChI_RDKit/Mol stereo mismatch	588	152	339
	Mol/Inchi/RDKit stereo mismatch	0	0	28
	RDKit_Mol/InChI stereo mismatch	23	22	1479
	Molecule has a bond with an illegal stereo flag	1054	0	0
	Molecule has a bond with an illegal type	6	0	0
	Molecule has a crossed bond in a ring	34	36	134
	Molecule has two (or more) atoms with exactly the same coordinates	4	5	2367
2	InChI_Mol/RDKit stereo mismatch	0	55	307
	Molecule has a stereo bond in a ring	2359	5763	7061
	Molecule has an atom with multiple stereo bonds	1493	52	3660
	Molecule has a stereo bond to a stereocenter	331	27	983
	Molecule has the 3D flag set for a 2D conformer	0	0	5
	Other InChI Warnings	20188	34052	170678
	No errors	15015	111137	177815

Note that the number of penalty scores output is not the same as the number of compounds as some compounds return multiple penalty scores

As expected, the ChEMBL literature set, being derived via manual extraction and curation, generates the fewest serious penalty scores, although it is clear there are still thousands of molecules with an undesirable representation (e.g. stereo bonds in rings, atoms with multiple stereo bonds and molecules with stereo bonds to stereocentres). The PubChem deposited set includes a large number of empty molfiles and unknown elements (often represented as an asterix, “*”) in addition to molecules containing overlapping atoms. The SureChEMBL set has a large number of compounds with V3000 formatted molfiles, radical entities as well as bonds with illegal stereo flags (usually molfiles with a 4 in the bond stereo field of the bond block). The difference between these sets is not surprising given the differences in the data sources, confirming that the *Checker* is suitable for identifying a range of different issues of differing severity for datasets of diverse origin.

A breakdown of the number of compounds from each set which returned penalty scores is shown in Table 3. When more than one score is returned for a compound the highest score is reported. It is worth noting that 75% of the ChEMBL literature set compounds and 60% of both the SureChEMBL and PubChem compound sets showed no issues in the *Checker* tests. When structures with a penalty score of 2 associated with InChI Warnings such as “omitted undefined stereo” and “InChI: Charges were rearranged” are excluded (as described above) then the proportion of compounds with undesirable features that would benefit from correction falls to between 4 and 11%.

**Table 3:** Percentages of the compounds in each of the SureChEMBL, ChEMBL and PubChem sets returning each value as their maximum penalty score

Penalty score	SureChEMBL	ChEMBL Literature	PubChem
7	0.01	0.00	0.45
6	1.62	0.00	3.14
5	2.72	0.15	1.00
2 (non InChI)	6.92	3.90	3.12
2 (InChI)	28.77	20.35	32.59
No errors	59.95	75.60	59.70

The highest (most serious) resulting score is the one recorded for each compound

From a ChEMBL perspective the compounds in the latest released version of the database (ChEMBL 26) have also been subjected to the *Checker* analysis and this has enabled the identification of small sets of compounds with particular problems that were not previously identified. This dataset is available as an sd file on the ChEMBL FTP site [38]. These have been prioritised for curation for a subsequent ChEMBL release. The numbers of compounds with penalty scores 5 and 6 are summarised in Table 4. Those considered to be the highest priority for curation are compounds with a penalty score of 6 (69 compounds). In future the structures of molecules with penalty scores of 6 will not be added to the database until they have been manually checked and corrected.

**Table 4:** Checker penalty scores on the current version of ChEMBL (ChEMBL 26)

Penalty score	Penalty explanation	No of compounds
6	InChI: Accepted unusual valence(s)	10
	Molecule has a radical that is not found in the known list	9
	Molecule has six (or more) atoms with exactly the same coordinates	50
5	InChI_RDKit/Mol stereo mismatch	810
	Mol/Inchi/RDKit stereo mismatch	6
	RDKit_Mol/InChI stereo mismatch	771
	Molecule has a crossed bond in a ring	632
	Molecule has two (or more) atoms with exactly the same coordinates	259

Compounds where the exclude flag is set are excluded from this analysis

The ~ 2500 compounds with penalty scores of 5 are placed in a group for second-tier curation with the following penalty annotations being prioritised:

- molecule has a crossed bond in a ring (632 compounds). This set does however need to be further divided depending on the ring size. It is considered a more serious issue for small rings than large rings where it is not always clear whether cis, trans or either orientation is correct.
- molecule has two (or more) atoms with exactly the same coordinates (273 compounds)

Those compounds with mismatches in the number of stereocentres will then be addressed.

### *Standardizer* component

**Fig. 2:**
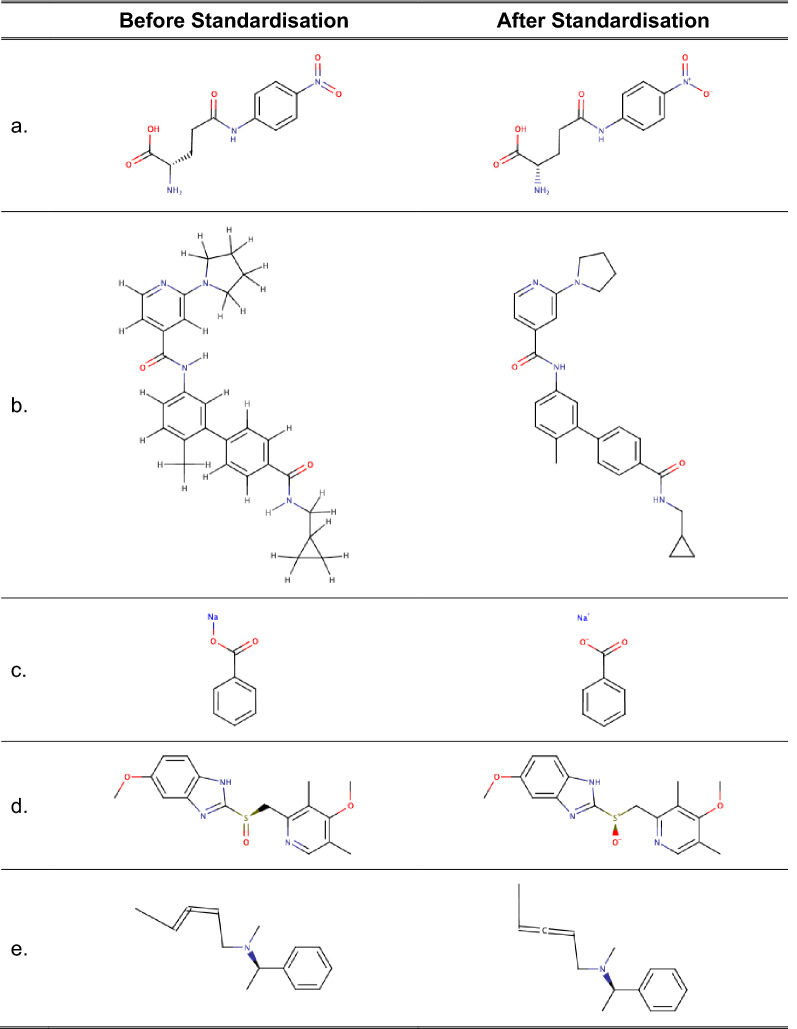
Examples of standardisations that have been applied to a set of compounds. The compound structure before and after standardisation is shown. **a** Fix hypervalent nitro groups, **b** remove explicit H atoms, **c** fix covalently drawn alkaline metals connected to O or N to ionic forms, **d** Standardise sulphoxides to charge separated form, **e** normalise (straighten) allene bonds

Examples of applying the *Standardizer* to some “ChEMBL-like” molecules are shown in Fig. 2. These examples show some of the specific standardisations described in the methods section for which the *Standardizer* component was designed to correct. Also, as described above, there will be differences between the last two versions of ChEMBL (versions 25 and 26) due to the re-standardisation as well as the manual curation that took place between the two releases. Comparing the structures in these two releases does not give any useful statistics on the effectiveness of the new *Standardizer*. Instead, to investigate this, the three sets of molecules previously described have been standardised by the new *Standardizer* module. The critical differences are where the new standardisation results in a different InChI from the one obtained prior to standardisation.

**Fig. 3:**
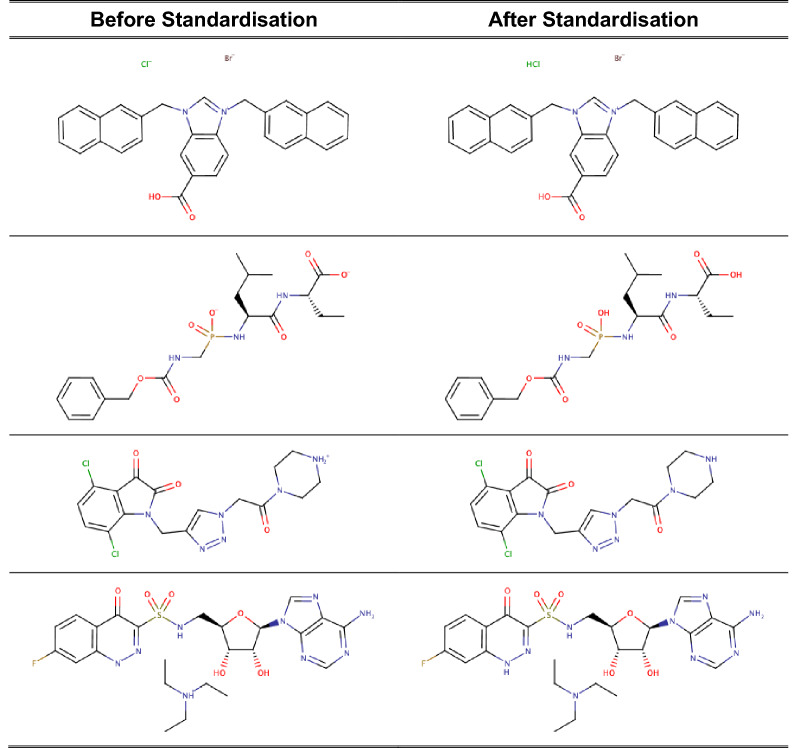
Examples of compounds from the ChEMBL literature set where the InChIKey changed on standardisation due to the rebalancing of the charge on the compound

The percentage of molecules with changed InChIs are low for all datasets and always < 2% in each case. The majority of the changes occur where the *Standardizer* has neutralised a previously charged molecule, for example by removing a proton from a positively charged nitrogen or protonating a carboxyl group. A summary of these changes is shown in Table 5. This also indicates which layer of the InChI has changed. Whilst initially it may appear that changes to the connectivity layer are unexpected for a standardisation process these examples are due to salts where the overall molfile is not neutral and where a proton has been removed to confer neutrality. To further exemplify this, some examples from the ChEMBL literature set are shown in Fig. 3 where the effect of standardisation on neutralising the molecules can be observed.

**Table 5:** Summary of the number of compounds that have changed InChIKeys following standardisation for the SureChEMBL, ChEMBL literature and PubChem deposited set

InChIKey layer change	SureChEMBL	ChEMBL Literature	PubChem
Connectivity	15	13	67
Connectivity and Protonation	5	1	33
Protonation	67	297	4358
Stereochemistry	11	0	16
Stereochemistry and Protonation	0	0	4
Total no of changed InChIKeys after standardisation	98	311	4478
Total no of compounds	520174	147008	297864
% changes InChIKeys	0.19	0.21	1.50

This also includes the number of compounds in the dataset and the percentage of the total sets with changed InChIKeys

**Fig. 4:**
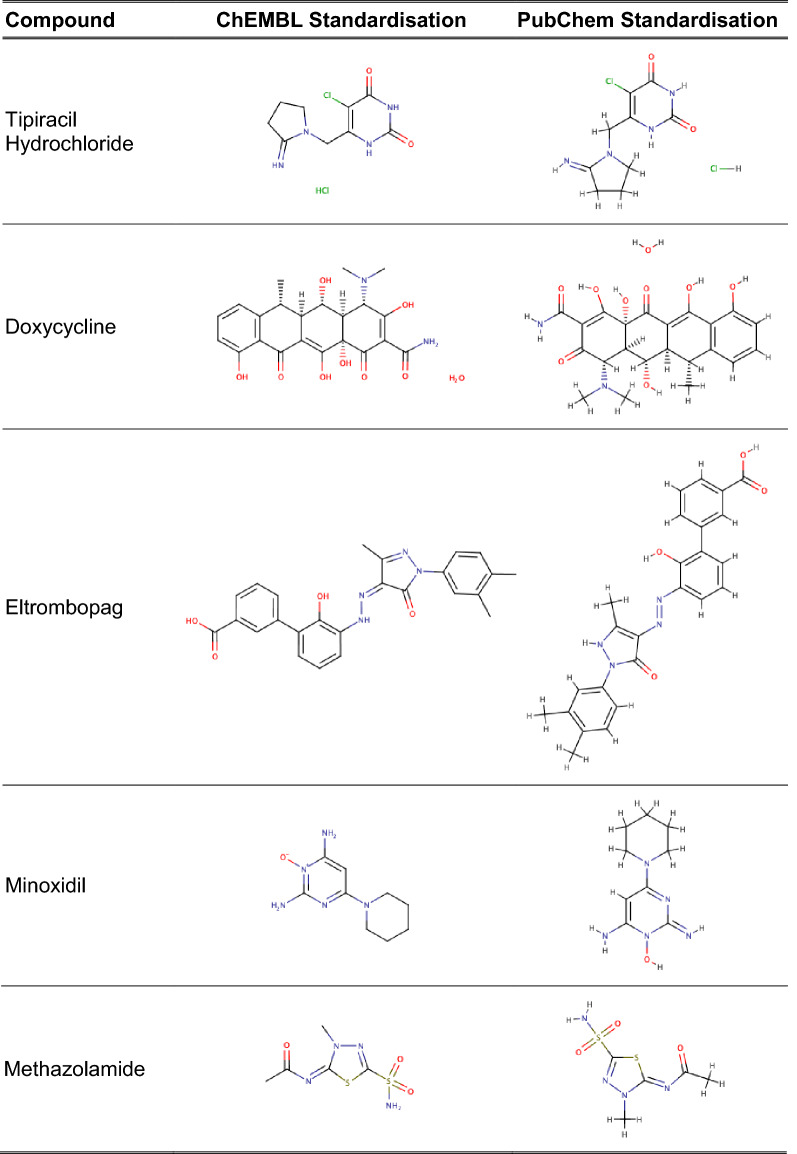
Examples of approved drugs standardised by the ChEMBL RDKit *Standardizer* and the PubChem standardiser

As different databases use different rules to standardise compound structures, it was of interest to compare the new ChEMBL *Standardizer* with the standardiser used by the PubChem database [39]. However, as the PubChem PUG service was slow to run via their API, only a small set of compounds were used for this comparison and comprised approved small molecule drugs as annotated in ChEMBL 25 (3071 compounds). This dataset is provided as an sd file in Supplementary Information S4. The ChEMBL 25 structures for these were taken and standardised using the new ChEMBL RDKit *Standardizer* and also the PubChem standardiser. In total there were 97 compounds that gave different Standard InChIKeys after the respective standardisations. The key difference is that the PubChem standardiser generates a canonical tautomer as part of the process but ChEMBL does not. In many instances this is not reflected by a change to the Standard InChI but for some molecules the InChI does change. There are also some differences in the double bond representation (cis or trans (either)). Furthermore, PubChem generates all structures with explicit hydrogens which are not present in the ChEMBL structures. Some of these examples are shown in Fig. 4.

### *GetParent* component

**Fig. 5:**
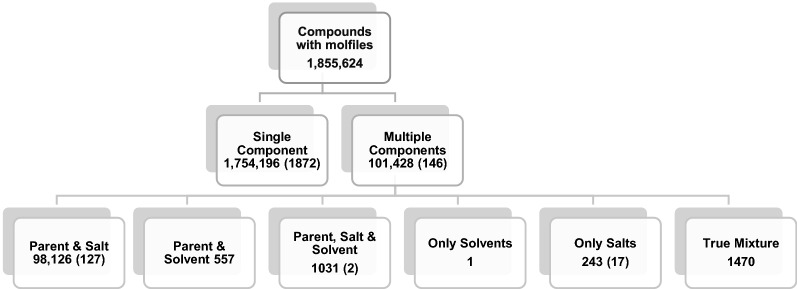
The composition and number of the compounds containing more than one component in ChEMBL 26 as identified by the *GetParent* module. The numbers in brackets refer to the number of compounds in each grouping that contain isotopes

The *GetParent* module was used to obtain the parent structures for the standardised structures from ChEMBL where these contained more than one component or an isotope. This resulted in over 100,000 compounds where a salt, solvent, isotope or combination thereof could be removed to create a parent structure. Molfiles that are a mixture of components can contain a number of different combinations such as: a parent and its salts; a parent, its salts and solvent; a combination of only salts; or a true mixture. A true mixture, for example, would include compounds such as Co-trimoxazole (CHEMBL58061) which is a marketed product comprising trimethoprim and sulfamethoxazole and which has been tested as a combination in a number of bioactivity assays. Additionally, any of these multicomponent compounds may have been tested for bioactivity or used in a clinical setting as a specific isotopic form. A summary of the composition of the multicomponent compounds in ChEMBL 26 are shown in Fig. 5. Whilst only ~ 6% of the compounds in ChEMBL are multicomponent (mostly salts), these compounds have over a million activity values recorded against them.

**Fig. 6:**
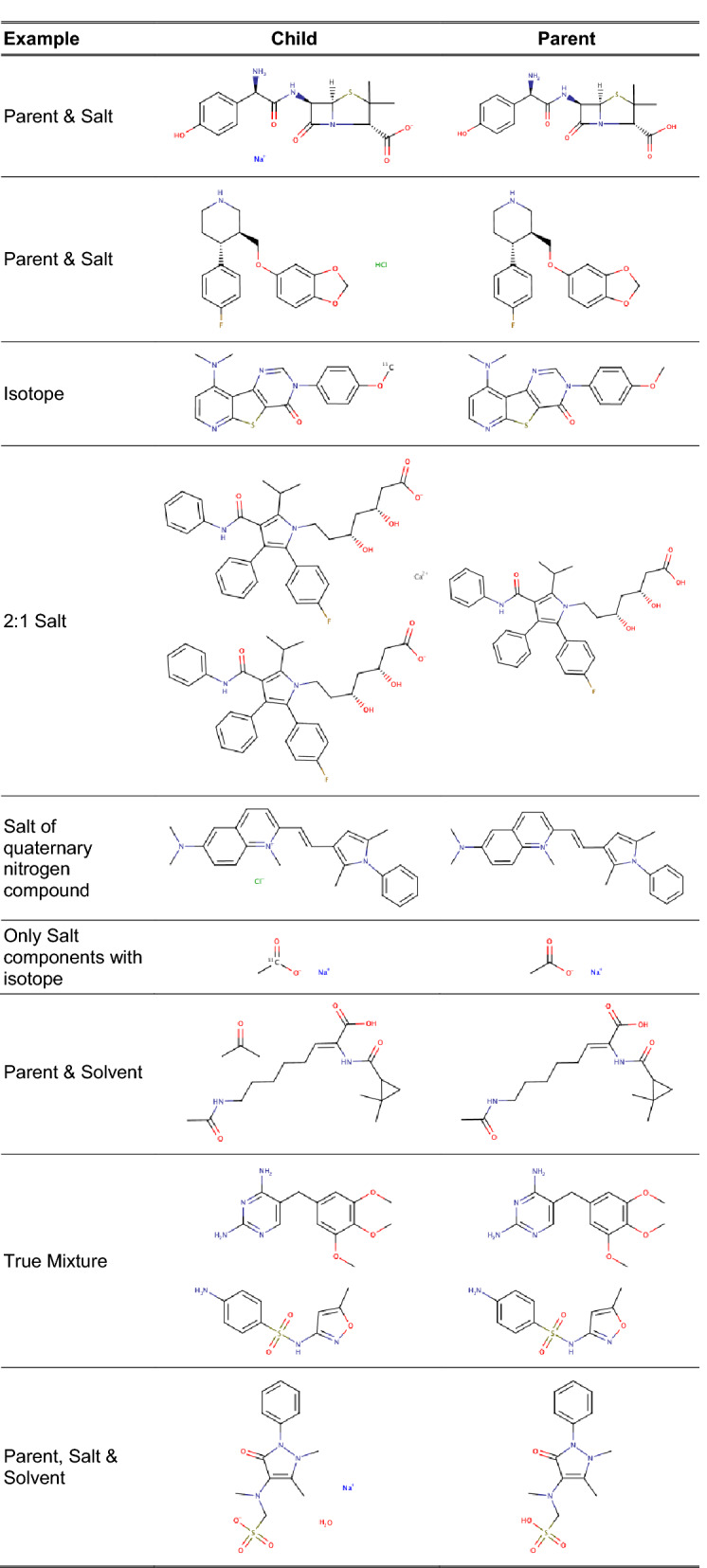
Examples of applying the GetParent module to some representative ChEMBL compounds containing varying combinations of salts, isotopes and solvents. The “Child” is the compound before and “Parent” the compound after the process has been applied

Some examples of the output from the *GetParent* process are shown in Fig. 6. This illustrates a number of features of the *GetParent* module including the need to re-standardise a parent to some extent once a salt is removed, and in particular to re-neutralise the charges on the parent molecule. The sodium salt of a carboxyl-containing compound is an obvious example of this. Once the Na+ cation is removed this leaves a net negative change on the parent so the *GetParent* module will add a proton to neutralise it. Quaternary nitrogen compounds are the exception, where the counterion (for example a chloride) is removed but, due to the quaternary nature of the nitrogen, it is not possible to neutralise the parent which therefore remains as the positively charged cation. Another important function of the *GetParent* module is that if a compound is a 2:1 complex with two parent molecules and one salt molecule, when the *GetParent* module is applied it can recognise that the two components remaining after salt removal are identical and so only one molecule is returned as the parent. Atorvastatin calcium is an example of this as can be seen in Fig. 6.

## Future work

Now that the components of the curation pipeline are being used in a production context for curation of compounds being added to the ChEMBL database, it will provide opportunities to review and refine any of the rules on the basis of a large consistently standardised dataset. The ChEMBL group will continue to evolve the curation pipeline as further needs are identified. The code is also now freely available to the community and other researchers are encouraged to suggest modifications so that the curation pipeline can improve further over time. Comments and issues can be added to the issues section of the ChEMBL Structure Pipeline GitHub repository [28].

## Conclusion

The three components of the structure curation pipeline described here have been developed as an open source project and are now available for researchers to use and adapt for their own applications. These components have been used by the ChEMBL group to produce the chemical structures in the latest release of the ChEMBL database (ChEMBL26). This has resulted in the correction of certain errors in standardisation that were present in previous ChEMBL releases and the identification of other issues that can now be prioritised for future manual curation. Additionally, the pipeline has been tested on less well curated datasets and demonstrated to be sufficiently robust to be used in the automatic structure checking and standardisation of such datasets. A comparison between the standardiser used by PubChem (another large publicly available database) and the one developed here gave only a small percentage of compounds with non-identical structures (defined as having different Standard InChIs). Where such differences were present this was largely due to variations in the business rules of the two database providers.

## Data Availability

The datasets used for the analyses are provided as sd files in Supplementary Files. S1_surechembl_set, S2_pubchem_set, S3_chembl_literature_set, S4_approved_drug_set and are available from the ChEMBL FTP site: ftp://ftp.ebi.ac.uk/pub/databases/chembl/standardiser_data_sets.
